# Cooperative Perception Technology of Autonomous Driving in the Internet of Vehicles Environment: A Review

**DOI:** 10.3390/s22155535

**Published:** 2022-07-25

**Authors:** Guangzhen Cui, Weili Zhang, Yanqiu Xiao, Lei Yao, Zhanpeng Fang

**Affiliations:** 1 Mechanical and Electrical Engineering Institute, Zhengzhou University of Light Industry, Zhengzhou 450002, China; c_u_i_guangzhen@163.com (G.C.); zwl8979@163.com (W.Z.); 2016008@zzuli.edu.cn (L.Y.); 2015073@zzuli.edu.cn (Z.F.); 2 Henan Province International Joint Laboratory for Intelligent Monitoring and Control of Complex Machinery and Equipment, Zhengzhou University of Light Industry, Zhengzhou 450002, China

**Keywords:** IoV, autonomous driving, cooperative perception, multi-sensor information fusion, DSRC, C-V2X, congestion control

## Abstract

Cooperative perception, as a critical technology of intelligent connected vehicles, aims to use wireless communication technology to interact and fuse environmental information obtained by edge nodes with local perception information, which can improve vehicle perception accuracy, reduce latency, and eliminate perception blind spots. It has become a current research hotspot. Based on the analysis of the related literature on the Internet of vehicles (IoV), this paper summarizes the multi-sensor information fusion method, information sharing strategy, and communication technology of autonomous driving cooperative perception technology in the IoV environment. Firstly, cooperative perception information fusion methods, such as image fusion, point cloud fusion, and image–point cloud fusion, are summarized and compared according to the approaches of sensor information fusion. Secondly, recent research on communication technology and the sharing strategies of cooperative perception technology is summarized and analyzed in detail. Simultaneously, combined with the practical application of V2X, the influence of network communication performance on cooperative perception is analyzed, considering factors such as latency, packet loss rate, and channel congestion, and the existing research methods are discussed. Finally, based on the summary and analysis of the above studies, future research issues on cooperative perception are proposed, and the development trend of cooperative perception technology is forecast to help researchers in this field quickly understand the research status, hotspots, and prospects of cooperative perception technology.

## 1. Introduction

Autonomous driving technology means that the vehicle utilizes sensors to collect information about its surroundings and process it in real-time to gain a better understanding of it. At the same time, the combination of location and mapping, path planning, decision-making, and vehicle control modules enables autonomous vehicles to drive on the road safely and efficiently without anyone taking over [1]. The implementation of autonomous driving will significantly mitigate the driver’s driving burden, improve energy efficiency, and reduce road safety traffic accidents.

The Society of Automotive Engineers (SAE) introduced the J3016 “Levels of Driving Automation” standard for consumers. The J3016 standard defines the six distinct levels of driving automation, including level 0 (no driving automation), level 1 (driver assistance), level 2 (partial driving automation), level 3 (conditional driving automation), level 4 (high driving automation), and level 5 (full driving automation) [2]. With the continuous development of autonomous vehicle sensor technology, it now plays a critical role in improving the sensing range and accuracy of autonomous vehicles, as well as ensuring that essential information required by autonomous vehicles while driving, including traffic signs, road information, obstacle information, and so on, is available [3,4]. However, autonomous vehicles above level 3 require continuous, real-time situational awareness of the surrounding environment [5], which is difficult to achieve by only relying on the perceptual ability of the vehicle itself for the following reasons:

- Insufficient environmental perception information. The perception module of autonomous vehicles primarily relies on numerous onboard sensors, such as LiDAR, cameras, millimeter-wave radar, etc. [6]. However, affected by various factors, such as sensor characteristics, obstacle occlusion, illumination, and bad weather, the vehicle’s perception range is limited, resulting in blind spots in the field of view and making it difficult to provide a full range of perception information for autonomous driving, which will cause autonomous vehicles to fail to detect imminent danger in a timely manner. For example, a Tesla Model X perception system mistakenly identified the white side of a tractor turning left in front of it as the sky, resulting in an accident in 2016 [7]. In 2019, a Tesla Model 3 Autopilot system driving at high speed failed to accurately identify the vehicle in front and perpendicular to itself and make braking decisions, resulting in a serious traffic accident [8].
- It is difficult for in-vehicle computing systems to process a large amount of multisource heterogeneous sensor data in real-time. There is no unified format for various sensor data types, and the fusion processing is complicated. In addition, for the object recognition network of an RGB camera, if the image resolution is 320 × 320 KB and the frequency of generating data is 50 Hz, the amount of data, in this case, will reach 14 MB/S [9]. The current method of processing massive amounts of data, usually equipped with high-performance computers for autonomous driving, will greatly increase the cost of autonomous vehicles. In addition, the diversity of multisource and heterogeneous sensor data formats will also increase the difficulty of data processing.

In order to make up for the insufficiency of autonomous driving perception capability and data calculation above level 3, advanced sensing technology, edge computing, communication, and other technologies need to be combined to build an autonomous driving cooperative perception system in the IoV environment, enhancing perception accuracy, improving the perception range, and reducing delay. Furthermore, this will provide more accurate and rich environmental information for autonomous vehicles in real-time and lay the foundation for the realization of autonomous driving above level 3 [10,11]. Simultaneously, cooperative perception in the IoV environment can reduce the number of onboard sensors, lower the cost of autonomous vehicles, and speed up the commercialization of high-level autonomous driving. Vehicle-to-vehicle (V2V) and vehicle-to-infrastructure (V2I) cooperative perception are the two main implementation methods, and the perception effect is also affected by various factors such as weather, obstacle occlusion, and computing power. At the same time, a large amount of multisource, heterogeneous sensor data must put forward higher requirements for communication performance.

**Figure 1:**
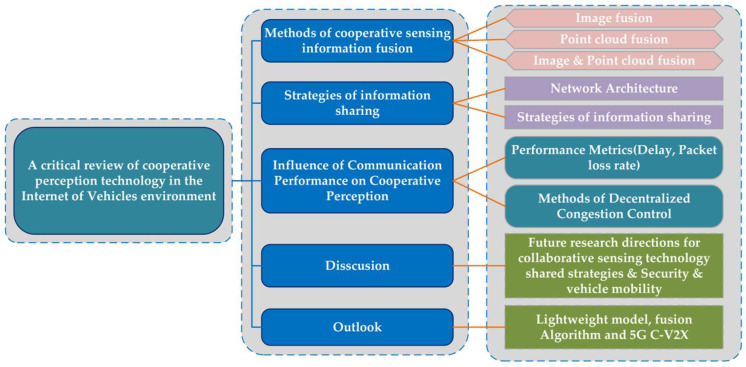
An overall diagram of the critical review on cooperative perception in the Internet of vehicles environment.

In the entire IoV cooperative perception system, this mainly includes the fusion, processing, and sharing of cooperative perception data. Among these, the multi-sensor information fusion method, information sharing strategies, and communication technology are the keys to realizing the cooperative perception of autonomous driving in the IoV environment. Multi-sensor information fusion is a prerequisite for cooperative perception. Compared to single-vehicle information fusion, cooperative perception multi-sensor fusion in the IoV environment has more abundant information sources, wider viewing angles, stronger adaptability, and can achieve higher perception accuracy and range [5,12]. Machine learning, as the mainstream technology of target detection, predicts and improves the perceptibility of the target by training the model, realizing the selective transmission of target perception information with the vehicle–road coordination device [9,13]. However, the existing research on cooperative perception information fusion methods is still in the stage of rapid development, and there are still deficiencies in real environment test applications and target detection research. Wireless communication networks play a bridge role in cooperative perception in the IoV environment, which is an important guarantee for information sharing. The quality of communication will directly affect the effect of cooperative perception. In addition, the cooperative perception information sharing strategy is also crucial to ensure the accuracy and timeliness of perception. Usually, the strategy of only broadcasting the target information that the autonomous vehicle cannot perceive is used to reduce the communication load, avoid the redundancy of perception information, and better optimize the balance point of perception–fusion–communication for autonomous driving cooperative perception in the IoV environment. Perception accuracy and end-to-end latency are two essential indicators for evaluating cooperative perception technology. Some researchers concluded that deploying a multiaccess edge computing (MEC) infrastructure could improve the effectiveness of cooperative perception and greatly reduce end-to-end communication delays by comparing the perception effect and transmission delay with a single intelligent vehicle and cloud-based computing [14,15]. Figure 1 provides an overall diagram of the critical review on cooperative perception in the IoV environment.

The paper is organized as follows. Section 1 introduces the background, significance, and related research status of autonomous driving cooperative perception technology in the IoV environment and points out that cooperative perception technology plays a key role in autonomous driving. Section 2 summarizes and analyzes the cooperative perception information fusion methods from three aspects, including image fusion, point cloud fusion, and image–point cloud fusion. Section 3 discusses the cooperative perception information sharing network, sharing strategy, and application-level communication performance indicators, and it analyzes and summarizes the impact of delay, packet loss rate (PLR), and channel congestion on cooperative perception during data transmission. Section 4 outlines the current challenges faced by cooperative perception by analyzing existing research and proposes recommendations for future cooperative perception in the IoV environment. Section 5 is the summary and outlook.

## 2. Cooperative Perception Information Fusion

**Figure 2:**
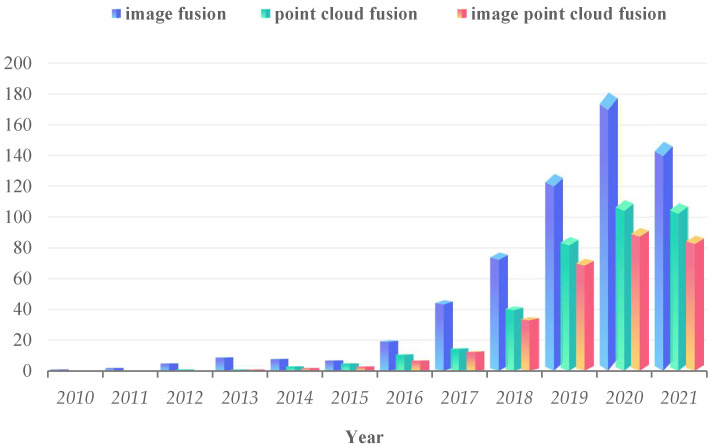
The increasing amount of research on cooperative perception multi-sensor information fusion in the Internet of vehicles environment.

Some breakthroughs have been made in the research of autonomous vehicle multi-sensor fusion methods, including image fusion, point cloud fusion, and image–point cloud fusion [3,16,17,18]. Although a multi-sensor redundant combination design can make up for the insufficiency of a single sensor in perception, reduce the uncertainty of target detection, and enhance the vehicle’s effective perception of surrounding environmental information, the test results in real scenarios are not ideal. For example, the fusion of camera and LiDAR can provide high-resolution image information and reduce the impact of lighting conditions, but the perception effect is still lacking in bad weather and obstacle occlusion. Furthermore, there are still many other problems to be overcome, such as imperfect information fusion methods, large amounts of data, limited network bandwidth, and spatiotemporal alignment problems. Figure 2 depicts the research trends of cooperative perception based on multi-sensor information fusion from 2010 to 2021. The data are from Google Scholar advanced searches of cooperative perception and different sensor fusion methods.

From the perspective of vehicle–road cooperative perception information fusion, this section summarizes the existing research on image fusion, point cloud fusion, and image–point cloud information fusion at different locations and from multiple perspectives. This section also briefly summarizes its existing challenges. Table 1, below, qualitatively summarizes the strengths and weaknesses of the commonly utilized perception-based sensors in AV based on their technical characteristics and other external factors, such as weather and illumination conditions. At the same time, it compares the perception effect of using only multi-sensor fusion and V2X fusion.

### 2.1. Image Fusion

Research on cooperative perception based on image information in the IoV environment started early and is relatively mature because the images have the advantages of high resolution and a good target classification effect, the research and applications of visual sensors are older, and the price is cheap. Image data is a common type of shared data between autonomous vehicles and other perception nodes.

The amount of raw video data collected by the camera is large among all kinds of environmental perception information, and the transmission of each video frame will cause the network to be overloaded, increase the transmission latency, and affect the timeliness of cooperative perception. In order to reduce the heavy network load caused by the amount of data, traditional image compression [19], deep learning [20], and other methods reduce the size of the transmitted image data to ensure the timeliness of cooperative perception. Löhdefink et al. [21] adopted image compression to reduce the size of data transmission. Combined with a lossy learning image compression method, the overall bit rate is reduced by an auto-encoder with an adversarial loss function [22], which effectively relieves pressure on the wireless communication channel. Different from traditional methods, Lv et al. [23] used the principle of separating static background and dynamic foreground, as well as the technique of video frame background removal and noise reduction calculated by pixel value, to quickly extract dynamic foreground from video frames. By using the generative adversarial network technique, reintegrating the static background and dynamic foreground into a new video frame, the correct driving decisions can be made based on the static background and dynamic foreground. According to the real dataset test, the method can reduce the transmission load of the video image data by more than 85 percent and decrease the processing time of perception information to 27.7% of the original on the premise of ensuring that important environmental perception information is not lost, effectively realizing the cooperative environment perception of autonomous vehicles and roadside infrastructure.

**Figure 3:**
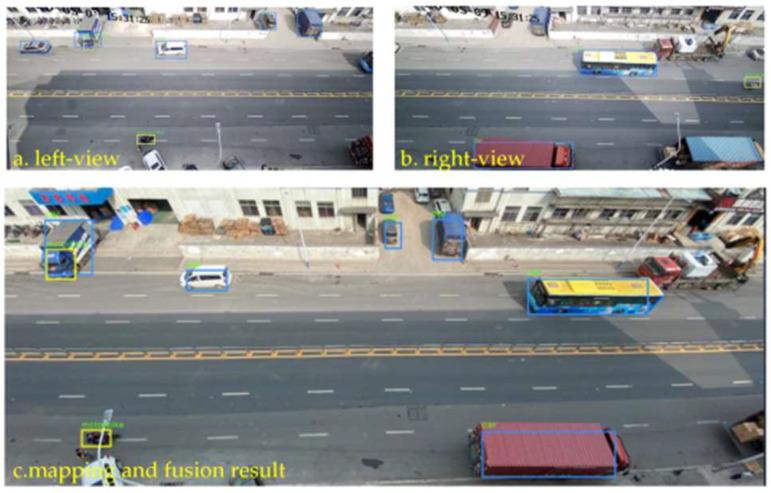
Object detection from different angles based on image fusion.

In addition, many scholars have studied the existing image fusion strategies for limitations and blind spots in the perceived field of view and range. Xiao et al. [24] used a deep learning method to extract key information from the vision system and expanded the perception range of the host vehicle by fusing the bird’s-eye view generated by the perception information of other vehicles in the vehicle network. This approach deepened the self-driving vehicle’s ability to understand its environment. Sridhar et al. [25] used cooperative relative positioning and map merging [26] to realize the cooperative perception of vehicles with a common field of view by sharing the image perception information of adjacent vehicles with the host vehicle. Liu et al. [27] used V2V communication technology, based on a 3D inter-vehicle projection model and selected feature point matching to estimate the geometric transformation parameters to achieve inter-vehicle image information fusion through deep-affine transformation. It effectively overcomes the problem of perceived blind spots in traffic jams, but it overlooks the impact of viewing angles, and, in some cases, there will be size deviations. If the perspective issue is taken into account, the scalable 5G multi-access edge computing-driven vehicle infrastructure cooperative system has a broader prospect. Through the distributed data fusion method, the environmental information detected by RSU cameras in multiple areas is interactively and perceptually fused. A global semantic description of the surrounding environment is formed, which greatly improves the perception range and accuracy of the vehicle [15], as shown in Figure 3. At the same time, the system introduces the high concurrency and low latency characteristics of 5G communication technology, which can effectively alleviate the communication load but lacks the evaluation of pose (such as position and direction). Table 2 summarizes the research status of image fusion in cooperative perception.

### 2.2. Point Cloud Fusion

The fusion of point cloud data from various angles and viewpoints has distinct advantages over the fusion of picture data in terms of the perception of the surrounding environment. LiDAR has the advantages of high spatial resolution, a wide detection range, and little influence from light. It plays an important role in the application of target recognition, ranging, and positioning in autonomous driving. This subsection describes and evaluates the available research in accordance with data-level, feature-level, and decision-level fusion categories [28] and focuses on the impact of abundant point cloud data and constrained network bandwidth on the efficacy of cooperative perception.

Data-level fusion research started early, with high precision but a large amount of data. This fusion method does not require any preprocessing of the data, and the heterogeneity of different data processing algorithms will not affect the accuracy of data being shared between vehicles. Chen et al. [29] were the first to suggest employing shared raw point cloud data to achieve cooperative perception, and they referenced a neural network for spare point-cloud object detection to detect objects in low-density point cloud data. This method effectively improves the perception range and detection accuracy of the vehicle. Simultaneously, it proves that existing DSRC communication technology can meet the data transmission requirements of point cloud fusion in the region of interest (ROI). Similarly, Ye et al. [30] introduced a state estimation framework for multivehicle LiDAR localization and sensor data fusion that utilized raw sensor data between different vehicles and achieved ground truth generation in an offline manner. At the same time, the registration method better matches point cloud data from diverse views, demonstrating that using perceptual data from multiple perspectives has a better auxiliary effect on cooperative perception, but that this increases the detection time.

Feature-level fusion refers to extracting feature information from original data and then performing fusion, which is currently the most studied and widely used fusion method, considering that the original point cloud data sharing will be limited by the bandwidth and delay of the wireless communication network. Based on a previous study [29], Chen et al. [31] innovatively proposed a point cloud feature-based cooperative perceptual fusion scheme, including a combined voxel feature fusion and spatial feature fusion method. This scheme can effectively improve the accuracy of perception while meeting the requirements of time delay and limited communication bandwidth. The results show that the detection effect at a range of 20 m is improved by about 10%, and the further distance is improved by about 30%. Wei et al. [32] used a multi-target tracking method [33] based on vehicle-mounted LiDAR and a voxel clustering algorithm to obtain the state of the surrounding environment. This method fuses the preliminary tracking results with the perception information of the RSU and other vehicles to generate the motion trajectory of the target vehicle. By using point cloud data from multiple objects, it can continuously operate under the condition of limited LiDAR perception or V2V communication failure, perceive the position of surrounding vehicles, ensure the stability of cooperative perception, and improve the accuracy of target tracking.

**Figure 4:**
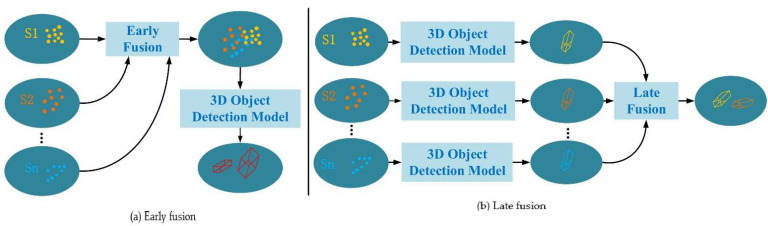
Early and late fusion schemes for cooperative 3D object detection.

Decision-level fusion refers to extracting the detected objects from each individual sensor and fusing the detection results. The fusion speed is fast and it is more suitable for data sharing, but there are few studies on it at present. Arnold et al. [34] proposed an early fusion (data-level fusion) and late fusion (decision-level fusion) 3D object detection cooperative perception scheme. This scheme adjusts the tradeoff between different fusion methods according to the sensor visibility and detection distance, selecting early fusion for high sensor visibility and short-range detection to ensure the detection effect and reduce the communication cost. On the contrary, if the visibility is poor and the point cloud data at the far position is small, late fusion is selected, and, finally, the two detected 3D bounding boxes are fused and shared, as shown in Figure 4. Experiments show that by fusing LiDAR data from different stages and positions of roadside units, spatial diversity with overlapping fields of view can be fully utilized to increase the density of point clouds, reduce false negative detection, more accurately estimate the bounding boxes of detected objects, and improve object detection accuracy.

In short, the multi-sensor data fusion method has a great impact on the timeliness and accuracy of cooperative perception. For example, sharing the original point cloud data has high sensing accuracy, but it will increase the detection time and transmission load, which cannot meet the low-latency requirements of cooperative perception. However, sharing the processed point cloud data can reduce the data transmission time, but the perceived accuracy will be reduced. Therefore, balancing the accuracy and real-time performance of perception is crucial to the effectiveness of cooperative perception. Table 3, below, summarizes the point cloud fusion methods classified by different fusion levels.

### 2.3. Image–Point Fusion

**Figure 5:**
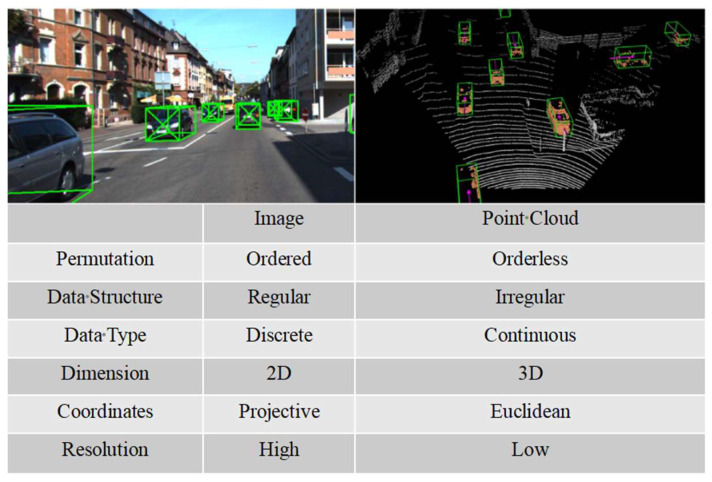
A comparison between image data and point cloud data.

Single-modal multi-sensor data fusion is always limited by intrinsic and extrinsic conditions. For example, the camera has a difficult time detecting the target depth information and is greatly affected by adverse weather and light. LiDAR has poor perception effects, low resolution, and a high cost for long-distance small targets. Millimeter-wave radars have strong antijamming capabilities but low resolution and a limited field of view. This subsection summarizes and analyzes the current sensor data fusion methods and challenges for single-vehicle autonomous driving [35,36] and points out that multimodal and multilevel sensor information fusion can greatly improve the effectiveness of cooperative perception. Figure 5 shows a comparison between image and LiDAR data characteristics.

Image–point cloud information fusion is the mainstream method of autonomous driving cooperative perception in the current IoV environment. Common fusion methods include data fusion estimation methods, based on the Kalman filter; the Bayesian-based distributed fusion method; and the neural network-based method [37]. Jiang et al. [38] proposed a robust target recognition algorithm based on the fusion of millimeter-wave radar and a camera based on the antijamming capability of millimeter-wave radar for foggy weather. The captured target is filtered by millimeter-wave radar and mapped onto the image to obtain an ROI. The detection results identified by the camera vision network are fused with the radar target estimation value using the weighted technique, resulting in perception results with greater detection accuracy. However, the method of fusing the perceptual information of radar and camera using convolutional neural networks has proved to be more promising. Ji et al. [39] used radar detection to create ROIs on images and utilized neural networks to classify the ROI, and similar object detection methods are also represented in the literature [40,41,42,43]. By fusing the results of the separate detection of the camera and the radar, Jha et al. [44] realized an effective correlation between the distance measurement of the radar and the image information. Deep learning methods based on generative adversarial networks used to achieve the fusion of camera and radar data have been widely studied [45]. Wang et al. [46] designed a radar and camera cooperative detection algorithm, projecting the radar signal to the image coordinate system through coordinate transformation and using the symmetry of the vehicle to identify the target vehicle, and they achieved good detection results. With the continuous deepening of research, some scholars have tried to deploy radar and camera perception equipment on the roadside to perceive the surrounding environment and provide more complete perception information for autonomous vehicles through wireless communication technology. Fu et al. [47] utilized edge computing to localize the environmental perception data to effectively reduce latency. Meanwhile, the YOLOv3 [48] and DBSCAN clustering algorithms [49], often deployed in roadside equipment, are used to preprocess camera and radar data, respectively, to obtain information such as the position, speed, and category of targets. Then, the method of joint calibration and direct update is used to synchronize the two types of sensors in space and time, and the Munkres algorithm and Kalman filter are used to achieve the association and tracking of multiple targets, which effectively improves the horizontal and vertical detection performance of the sensing system but lacks real-world verification. Wang et al. [50] realized the fusion perception of radar–camera sensors in a real road environment, which reduced the delay of actively perceiving the target and accelerated the actual deployment of cooperative perception technology.

In addition, some studies have compared the perception effects of different sensor combinations and found that the data fusion of cameras and LiDARs has richer environmental information and better target detection performance. However, due to the difference in the number of wire bundles and the detection distance, the amount of environmental information data collected by LiDAR is very different, which will have a certain negative impact on the timeliness of cooperative perception and the accuracy of target recognition. Satio et al. [51] proposed a LiDAR–camera sensor fusion method for long-distance point cloud sparse problems. Duan et al. [4] proposed an image–point cloud-based cooperative perception system, which mainly solves the problem of blind spot perception with intelligent sensors of AV at intersections. The roadside terminal is used to send the global map of the laser point cloud to the autonomous vehicle. In turn, the vehicle sends its position to the RSU. Finally, the RSU sends the perception result of the intersection to the vehicle. The experimental results show that the method improves the perception range and accuracy of vehicles at intersections. However, the real-time performance of perception is poor. References [52,53] utilize image and point cloud fusion algorithms for better environmental perception. The final decision is made by the telecommunication control unit by extracting objects from the environment that affect the multi-channel V2X communication system. In addition, an attempt is made to solve the problem of communication performance degradation due to the surrounding environment of V2X communication vehicles to improve the stability of multi-channel telecommunication control units. Gu et al. [54] achieved a breakthrough in the camera–LiDAR fusion strategy to cope with different scene changes. By cascading point clouds and images to generate fusion networks, including single-modality modes of laser point clouds only and multimodality modes of laser point clouds and camera images, it dynamically responds to more environmental changes and achieves higher perceived accuracy. Table 4 presents the existing research on image–point cloud fusion methods.

In addition, the spatial and temporal alignment of sensors is a prerequisite for information fusion in the sensor information fusion process. Spatial alignment considers the coordinate transformation of the targets of sensor data from different sources. In contrast, temporal alignment mainly focuses on how to solve the problem of the time difference between sensing information collected from edge nodes and received by other vehicles. Spatial alignment is often based on a constant turn rate and acceleration motion model. Temporal alignment typically transforms object coordinates using unscented transformations. Based on these two key problems, research on spatial and temporal alignment in multi-sensor fusion is equally important [55,56,57]. Allig et al. [55] summarized and analyzed the existing alignment methods, proposed using the unpredicted sender state for transformation, and compared it with the existing coordinate transformation method using the compensated sender motion prediction. Experiments show that the former has more obvious advantages, which not only reduces the computational complexity but also has more accurate state estimation. In multi-sensor perception data fusion in advanced fusion architecture, each sensor and each vehicle will preprocess their raw data, and the temporal alignment and spatial alignment in the post-data fusion process are essential factors to ensure the accuracy of the results [58].

### 2.4. Summary

Based on the existing literature, this section summarizes the current research status of cooperative perception technology for autonomous driving in the IoV environment from the perspective of cooperative perception information fusion methods, focusing on summarizing image fusion, point cloud fusion, and image–point cloud fusion methods. By comparison, we found that the fusion of image point clouds under multimodality and multi-view perspectives is the best method for cooperative perception (as shown in Table 3). Compared to single-vehicle multi-sensor fusion, cooperative perception information fusion has more advantages in terms of sensing range, accuracy, and reliability. Comparing the information fusion at different stages, the fusion and sharing of the processed data can not only reduce the amount of data transmitted but also significantly reduce the network communication load. The fusion and sharing of the original data are better for cooperative perception, but they will increase the communication burden and have a certain negative impact on the cooperative perception results. In the future, direct and effective cooperative perception fusion algorithms, and the interactive fusion of distributed sensor data, will become research trends. At the same time, the dataset construction of vehicle–road cooperation will be a key factor in ensuing research on and evaluation of vehicle–road cooperation algorithms. Table 5 shows a comparison of different sensor fusion methods.

## 3. Cooperative Perception Information-Sharing

Vehicle-to-everything is an important guarantee for realizing the cooperative perception of automatic driving in the IoV environment. Information interaction between autonomous vehicles and edge nodes is the basis for realizing cooperative perception. The transmission of cooperative perception messages (CPMs) between vehicles and edge nodes requires a certain bandwidth and has strict delay constraints, which sets higher requirements for network performance. Vehicle mobility and market penetration have a great negative impact on cooperative perception effectiveness and network communication quality. In addition, a reasonable CPM-sharing strategy is also very important, which defines how often vehicles share perception data and which perception data to share. Therefore, reasonable network communication technology and sharing strategies are necessary. Usually, it is necessary to select an appropriate communication network and set a reasonable sharing strategy according to different communication and traffic scenarios.

### 3.1. Cooperative Perception Information-Sharing Network

Currently, there are two main V2X communication technologies, including dedicated short-range communication (DSRC) and cellular vehicle-to-everything (C-V2X) [59]. Among them, DSRC [60] is specifically used for V2V and V2I communication. It has the advantages of a high transmission rate, low latency, and support for point-to-point and point-to-multipoint communication. C-V2X communication, represented by 5G, has the advantages of wide coverage, high reliability, and large data capacity. It is suitable for vehicle–road coordination and communication between edge servers. These two communication modes jointly support the diverse application requirements of the IoV environment.

#### 3.1.1. DSRC

One of the keys to enabling wireless communication technology standards designed specifically for vehicular communication is DSRC [61]. The DSRC communication protocol in the United States is based on the IEEE 802.11p and the IEEE 1609 series standards, which are collectively known as wireless access in vehicular environments (WAVE) standards [62]. Similarly, the European Telecommunications Standards Institute (ETSI) has developed the ITS-G5 protocol based on the IEEE802.11p standard to support the high quality of service requirements of autonomous driving applications [63]. Wei et al. [64] compared and analyzed the characteristics of wireless local area networks, WAVE, and 4G networks and proposed using a neural network model to select an appropriate communication method automatically and dynamically according to a vehicle’s density, speed, and data volume before data transmission. However, in recent years, studies have found that DSRC is not enough to support reliable and efficient V2X applications, especially in the case of high vehicle density and highspeed vehicle movement, the communication performance will be significantly reduced [65]. To improve its scalability and quality of service and to make up for the lack of performance in high-mobility environments, a new study group called IEEE 802.11 Next Generation V2X was formed in March 2018 [66]. This resulted in the formation of the IEEE Task Group 802.11bd in January 2019.

#### 3.1.2. C-V2X

**Figure 6:**
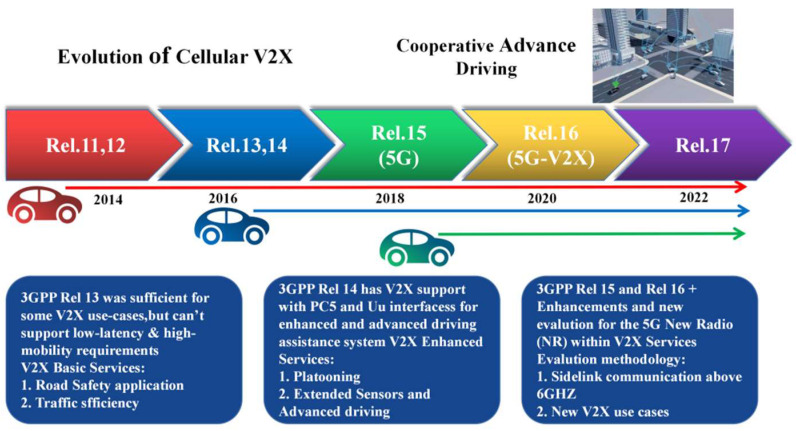
C-V2X standardization and evaluation.

C-V2X was standardized by the 3rd Generation Partnership Project (3GPP), which includes long-term evolution (LTE)-V2X and 5G-V2X (NR) technologies and is gradually enhanced through the evolution stages shown in Figure 6 [67,68]. In addition, C-V2X defines a PC5 interface that enables direct V2X communication through the sidelink and a Uu interface that realizes communication between the terminal and base station utilizing the uplink/downlink. It has high network capacity and wide coverage and can better support autonomous driving cooperative perception in the IoV environment, improve the reliability of data transmission, decrease transmission delay, and reduce frequent horizontal switching in the network. C-V2X offers performance advantages over DSRC in terms of its additional link budget, higher resilience to interference, and better non-line-of-sight (NLoS) capabilities [69]. Based on [65], Choi et al. [70] further analyzed this concept based on DSRC and 4G cellular networks, which are not enough to support the large-scale sharing of raw sensor data. Three types of vehicle networks, including 5G cellular, a modified version of IEEE 802.11ad, and a dedicated new standard, were proposed. In addition, it was pointed out that these three communication methods can effectively avoid the channel congestion problem caused by a large amount of data transmission. On the other hand, they can improve the real-time performance of the vehicle networking perception system. At present, C-V2X is in the actual deployment and application test stage, and the system is gradually improving. The latest release of New Radio (NR) V2X was specified in Release 16 (Rel-16) as a complementary access technology defined to better serve sophisticated applications and use cases with more stringent requirements (e.g., platooning, advanced driving, etc.). Meanwhile, the standardization and testing work of Rel-17 is actively promoted [71].

#### 3.1.3. Hybrid Architecture

**Figure 7:**
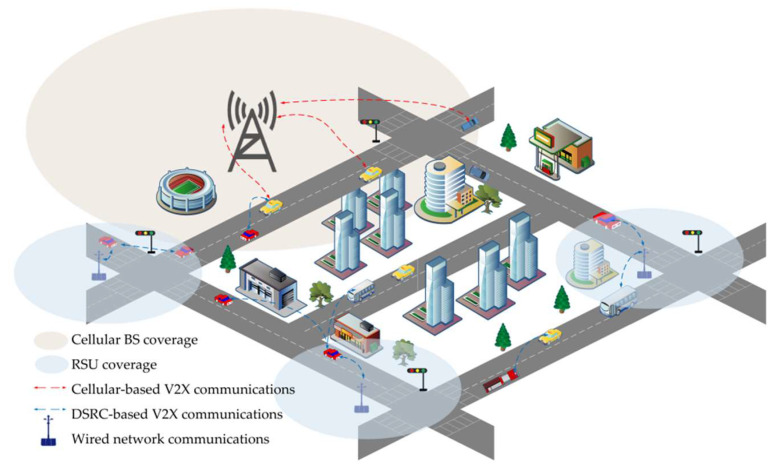
V2X communication in a DSRC–cellular hybrid urban scenario.

At present, there are obvious limitations for a single V2X technology to achieve efficient and reliable cooperative perception in the IoV environment, such as the insufficient penetration rate of smart devices, network switching caused by vehicle mobility, and limited network bandwidth. To this end, some researchers have proposed that a hybrid solution using DSRC and C-V2X technology is more feasible for actual deployment at this stage, as shown in Figure 7 [72]. This hybrid architecture enables the cellular network to back up vehicle data when the V2V multi-hop connection in the sparse network is interrupted. Meanwhile, it can choose the communication mode independently according to the driving scene requirements and performance requirements, which is more in line with economic development and accelerates the commercial application of autonomous driving. Liu et al. [73] conducted an in-depth analysis of the potential of DSRC and cellular network hybrid architecture [74,75], which fully considered the limitations of the two current mainstream V2X communication technologies in supporting V2X applications and the challenges brought by vehicle mobility to wireless communication. It concluded that the hybrid architecture of DSRC and cellular networks is promising for realizing V2X applications with low latency and high-reliability requirements.

**Figure 8:**
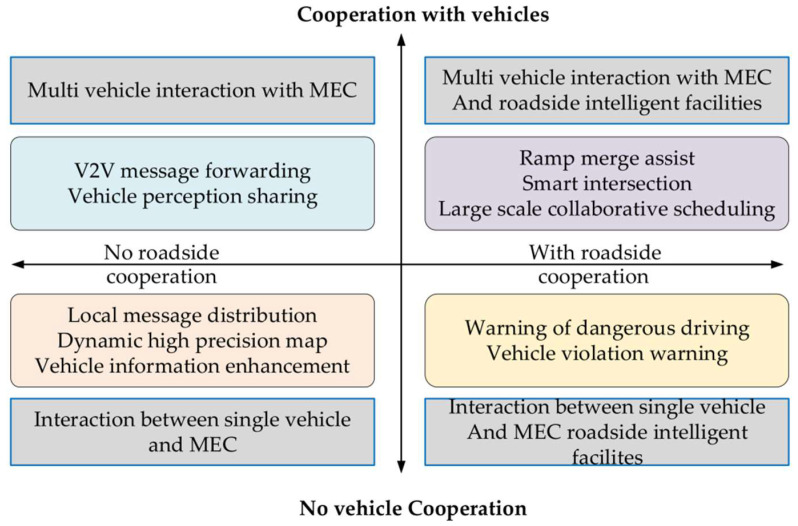
Application scenarios for 5G and C-V2X.

In practical application scenarios, to solve the problems of high network transmission latency and reduced reliability in cases of highspeed vehicle driving, Zhu et al. [76] proposed a 5G C-V2X intelligent fusion network technology based on MEC, and they discussed and summarized the network architecture, deployment schemes, and application scenarios. As shown in Figure 8, according to the needs of vehicle–infrastructure collaboration, the application scenarios of the intelligent fusion network are divided into four categories: interaction between a single vehicle and the MEC; interaction between multiple vehicles and the MEC; interaction between a single vehicle and the MEC or roadside intelligent facilities; and multivehicle interaction with the MEC or roadside smart facilities. In addition, the integrated network architecture of MEC and C-V2X effectively shortens the transmission path of data services in the IoV environment with localized services and realizes the information exchange delay to meet the service requirements of ultra-high reliability and low latency in the C-V2X scenario. In addition, the differences in the driving speeds of autonomous vehicles also require different performance requirements for network communication. When the vehicle speed is too high, it will pose higher challenges to edge node sensing data, computing resource allocation, and task scheduling. Fukatsu et al. [77] pointed out the importance of using millimeter-wave communication in cooperative perception in combination with the requirements of autonomous vehicles for network data transmission at different driving speeds. As the speed of the vehicle increases, the data rate generated by the sensor increases exponentially, and they pointed out that, when using V2V millimeter wave communication on the 60 GHz band, the cooperative perception of overtaking scenarios can still be achieved when the speed exceeds 51 km/h. Fukatsu et al. [78] studied the sensor rate required for cooperative perception to achieve specific application functions for urban road scenes, and they found that millimeter-wave communication showed better performance in realizing the cooperative perception of edge nodes. It was verified that, when vehicle speeds are 56 km/h and 47 km/h, the required data rates are 12 GHz and 6 GHz, respectively.

**Figure 9:**
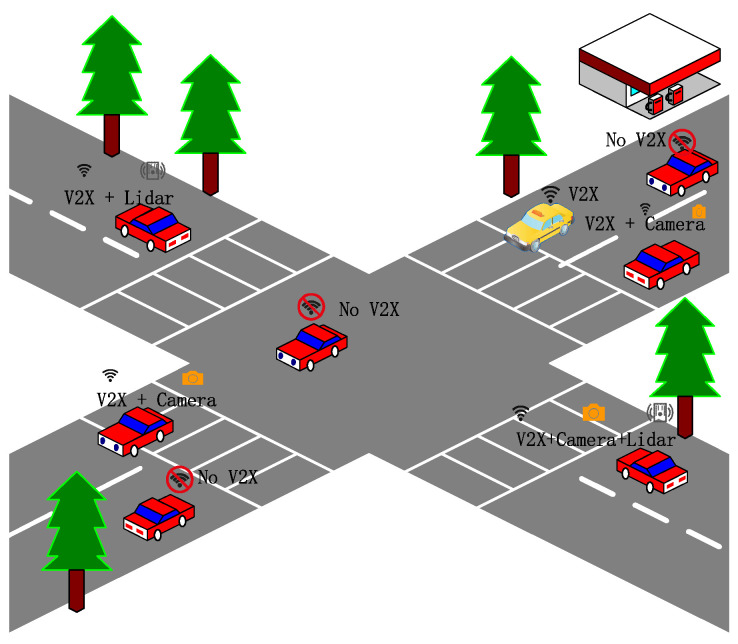
Illustration of the cooperative perception conception.

At present, the penetration rate of intelligent networked vehicles is low, and the deployment of intelligent equipment and infrastructure construction still need continuous efforts. Therefore, market penetration is an important factor that cannot be ignored for synergistic perception effects and communication quality. When the penetration rate is too low, the communication link supporting data transmission may be unstable due to the distance, and it may even be difficult to match a suitable edge node. Radovan et al. [79] analyzed different combinations of vehicle V2X and sensors and pointed out that, in the case of a low penetration rate of smart vehicle market deployment in the early stages, different sensor and communication equipment deployment schemes will effectively improve the ability of cooperative perception. As shown in Figure 9, each vehicle is equipped with different configurations and quantities of communication equipment and sensors. Using cooperative perception information interaction, the impact of the low penetration rate can be effectively reduced and the range of perception can be improved. Li et al. [80] studied the accurate estimation and prediction of traffic system microstates based on the particle filter cooperative perception framework and found that the market penetration of connected autonomous vehicles has a great influence on the estimated values and predictions. When the market penetration rate is 50%, the estimated accuracy of vehicle positioning and speed is 80–90%.

Wang et al. [81] analyzed the demand for V2V and V2I network capacity to realize cooperative perception under different traffic densities and penetration rates. When the proportion of autonomous vehicles to the total number of vehicles is too low, it is difficult to find V2V links that support data because of the distance between autonomous vehicles. Under the conditions of limited penetration and reliability requirements, V2I communication can be used for data transmission. Then, the V2I flux from the CPM exchange was analyzed to be highest at medium permeability (0.5). Table 6, below, summarizes the existing research on cooperative perception information-sharing and communication technology.

To sum up, the cooperative perception sharing network is crucial for improving the perception capability of intelligent connected vehicles. The deployment and application testing of wireless communication technology at this stage have achieved preliminary results. Vehicle mobility and market penetration have a great impact on the effectiveness of cooperative perception and the quality of network communication at this stage. When the penetration rate is too low, the communication link supporting data transmission may be unstable due to the distance, and it may even be difficult to match a suitable edge node. At the same time, the problems of high network transmission delay and reduced reliability caused by highspeed vehicles are also the focus of our attention.

### 3.2. Cooperative Perception Information-Sharing Strategy

The cooperative perception information-sharing strategy plays a key role in alleviating network load and enhancing the cooperative perception effect. If the cooperative sensing information is shared too frequently, the wireless channel will be overburdened, resulting in channel congestion. It will lead to the perception information age being too old, reducing the accuracy of perception. Similarly, sharing all sensory data will result in information redundancy, leading to wasted computing and communication resources. If part of the perception information is shared, the perception will be incomplete, and the accuracy and integrity of the perception will be reduced. Therefore, a reasonable sharing strategy is particularly important for autonomous driving cooperative perception, including how often vehicles share perception data, which perception data to share, and the format of the shared data.

For research on perception information-sharing strategy, the ETSI recently approved a technical report, which is the first proposal to standardize the CPM and the CPM generation rules [82]. The CPM generation rules define the frequency of vehicle generation and the transmission of CPM as well as the content of CPM, including onboard sensors (detection range, field of view, etc.) and detection targets (position, speed, size, etc.) [83]. The current ETSI CPM generation rules establish that a vehicle has to check every T_GenCpm if a new CPM should be generated and transmitted, with 0.1 s ≤ T_GenCpm ≤ 1 s (100 ms by default). However, these generation rules are preliminary and only a first proposal (hence subject to possible changes). A vehicle should generate a new CPM if it has detected a new object or if any of the following conditions are satisfied for any of the previously detected objects:

1. Its absolute position has changed by more than 4 m since the last time its information was included in a CPM.
2. Its absolute speed has changed by more than 0.5 m/s since the last time its information was included in a CPM.
3. The last time the detected object was included in a CPM was one or more seconds ago.

Thandavarayan et al. [84,85] compared the dynamic CPM generation strategy formulated by ETSI with the periodic generation strategy (the periodic generation strategy is that the vehicle periodically broadcasts the information from all detected objects). The results show that the dynamic CPM generation strategy is more flexible than the systematic generation strategy, which effectively reduces the transmission of low-value data. Furthermore, on this basis, the dynamic generation CPM strategy is optimized. In the proposed optimization strategy, when CAV receives updated information about an object from other vehicles, the vehicle does not repeat the broadcast of the object’s information, further reducing information redundancy and decreasing communication overhead.

To undertake cooperative perception comparisons and investigate the relationship between communication bandwidth and the amount of transmitted data, Kim et al. [26] used raw sensor data, processed sensory data, and compressed data. The results reveal that as the amount of data communicated increases, so does the communication latency. Instead of exchanging raw sensor data, Rauch et al. [86] analyzed the influence of communication delay and transmission range on cooperative perception and proposed that the shared detected object data strategy is more time-effective. The majority of data transmission methods are now provided to the target vehicle via broadcasts. If an appropriate coordination mechanism is not in place throughout the transmission process, it will cause data transmission confusion, raise the likelihood of data retransmission, diminish transmission efficiency, and increase the likelihood of packet loss, all while raising network channel load. Baldomero et al. [87] addressed the problem of [86] by using a context-based message acknowledgment method. The mechanism takes advantage of the fact that the transmitting vehicle can request the confirmation of certain or crucial broadcast information and retransmit anomalous broadcasts on a selective basis. The approach lowers data transmission interference and collisions while also increasing data transmission accuracy.

Furthermore, many scholars define CPM-sharing strategies according to the value of CPM [88,89]. Higuchi et al. [90] proposed deciding whether to send the CPM policy by predicting the importance of the CPM to the receiver. If the detected object information is included in the cooperative perception information, it is transmitted only when the sender evaluates the object to be valuable to the receiver. This method reduces the information transmission rate of low-value objects and realizes the effective control of channel congestion. However, the difficulty is how to obtain an accurate estimate of the information value. This problem was partially solved by [91], who proposed selectively transferring higher-valued data of perceived demand using reinforcement learning. Reference [92] proposed enhancing the timeliness and accuracy of cooperative perception information by improving the freshness of sensing information. Talak et al. [93] defined the coverage of the ROI of the vehicle and the update rate of perceived information. When communication resources are limited, they choose to maximize the perceptual data of the ROI of the shared vehicle. At the same time, based on the premise of ensuring the most appropriate CPM update frequency, the information of interest is selectively transmitted. This can expand the perception range, reduce the information age, and achieve real-time situational awareness of the surrounding environment.

With the continuous development of intelligent roadside equipment, the interaction of information between the vehicle and the roadside is equally important to enhance the cooperative perception effect. The RSU is usually located in a fixed location and supports the deployment of edge computing devices, so it can better support the perception data processing capability of autonomous driving. By analyzing the advantages and applications of existing V2I communication systems, Malik et al. [94] pointed out the important role of V2I communication in realizing autonomous driving cooperative perception. Noh et al. [95] proposed a cooperative system utilizing V2I communication. It can combine its perception data with a high-precision map to assist the self-driving vehicle in better understanding the driving environment and provide driving suggestions when the vehicle enters the service range of the roadside equipment. The performance of the proposed cooperative system was tested in two traffic scenarios of road icing and construction, which proved the effectiveness of the cooperative perception of the system. Perceptual information-sharing strategies are summarized in Table 7 according to the type of shared information.

### 3.3. The Effect of Network Performance on Cooperative Perception

The realization of cooperative perception relies on the interaction between V2X communication and the perception information of the surrounding environment, which will significantly increase the amount of information exchanged by vehicles. At the same time, problems such as unstable communication quality and channel congestion are prone to occur in the process of information exchange, resulting in a series of negative effects such as high transmission latency and packet loss, which reduce the effectiveness of cooperative perception. In fact, the loss and latency of data packets in the real driving environment are inevitable, and cooperative perception also often uses latency and PLR as evaluation indicators for the application of cooperative perception technology. We compare the impact of PLR, latency, and channel congestion on cooperative perception in this subsection, summarizing and analyzing relevant research in recent years.

#### 3.3.1. Latency

Latency in V2X cooperative perception includes task offloading latency, data fusion calculation latency, and result feedback latency. The size of the computing task, the bandwidth of the wireless channel, and the computing power of the edge server or mobile device all have a certain impact on latency.

#### 3.3.2. Packet Loss Rate

The PLR is the ratio of the data packets lost by the target node to the data packets sent by the application layer of the source node. Data integrity is very important for the effectiveness of cooperative perception in the process of data transmission. In fact, under the interference of multiple factors, such as unfavorable weather, illumination, and communication quality, it is difficult to avoid the loss of perception data during transmission. If the data loss is serious, it will reduce the reliability of perception results and even lead to the failure of cooperative perception.

#### 3.3.3. Congestion Control

The decentralized congestion control (DCC) mainly focuses on solving the problem of channel congestion in cooperative perception information sharing and improving the timeliness and accuracy of cooperative perception information. Currently, DCC technology mainly reduces the congestion of the communication channel by adjusting the generation strategy of cooperative sensing information and controlling the transmission parameters. Among these, adjusting the generation strategy of cooperative perception information mainly reduces the redundancy of cooperative perception information by optimizing the message generation rules. The most common control parameters are transmission rate control, transmission power, and transmission data rate control [96].

The safety-critical application of autonomous driving has strict constraints on the delay, and the time interval delay between the generation and transmission of cooperative perception cannot exceed 100 ms [82]. The pros and cons of communication performance have a direct impact on the effectiveness of cooperative perception, which, in turn, interferes with the execution of decision-making and control modules [97,98]. When the communication delay and data PLR in the communication process reach a certain level, it will have a great negative impact on the use of V2X information in autonomous driving, resulting in a poor cooperative perception effect, which, in turn, reduces the safety of autonomous driving. In order to analyze and reduce the negative impact of delay and PLR on network performance, and, thus, improve the effectiveness of cooperative perception, domestic and foreign scholars have carried out much research on this. Liu et al. [99] constructed several typical IoV application scenarios in a closed test field environment for the needs of DSRC test evaluation. The experiment used PLR and delay as evaluation indicators and tests and analyzed the effects of speed, distance, shelter, and other factors on the performance of DSRC. The test results show that communication distance and obstacle occlusion are the main factors that cause the degradation of DSRC communication performance. Obstacle occlusion increases the PLR of communication, resulting in security information not being fully transmitted under NLoS conditions, reducing the effectiveness of cooperative perception. Similarly, Bae et al. [100] designed and implemented a performance evaluation of DSRC communication in both Line-of-sight (LoS) and NLoS scenarios in real scenarios. When the transmission power is 5 dBm and the packet reception rate exceeds 90%, the communication distances in the LoS and NLoS test scenarios are 720 m and 175 m. When the transmission power is 11 dBm and the packet reception rate exceeds 90%, the communication distances in the LoS and NLoS test scenarios are 1035 m and 515 m. Through comparative analysis, communication distance and occlusion can have a certain impact on the packet reception rate, which, in turn, affects the effectiveness of perception.

Xu et al. [101] used performance indicators such as PLR, latency, and throughput to evaluate the performance of communication devices under high channel occupancy in dense traffic scenarios. They proposed that a high channel occupancy rate will obviously lead to a decline in vehicle communication capabilities and cannot fully guarantee the effective transmission of cooperative sensing data. Lee et al. [102] comprehensively considered the impact of packet loss and delay on V2X data fusion, implemented automotive augmented reality by integrating V2X communication and information from the vehicle itself, and evaluated the impact of sensor fusion with packet loss and delay [103]. In the experiment, when the packet loss in the lossy network was set to 5% and the delay was 1 s, the accuracy of data fusion was nearly doubled compared to normal. When the data packet loss in the communication network reached 5%, broadcasting and receiving messages one time per second, the accuracy of data fusion was about 37% lower than normal. Xiong et al. [104] analyzed the impact of packet loss and delay on the blind-spot pedestrian collision system. Under a certain initial longitudinal distance, the higher the PLR, the lower the security, and the higher the initial speed, and the smaller the limit delay.

In addition, the performance of V2X communication is highly affected by the communication load, and high channel load levels increase the risk of packet collisions. The vehicle network integrates congestion control algorithms to control the channel load and avoid congestion, mainly including reactive and adaptive methods [105]. These protocols can modify the rate or power of information transmission or even drop packets, changing the transmission of V2X messages, which, in turn, affects the effectiveness of cooperative perception. Günther et al. [106] analyzed the impact of the ETSI ITS G5 network on cooperative perception, including the generation rules of cooperative perception information [84] and the influence of broadcast frequency on communication channels. They evaluated the impact of DCC on cooperative perception by defining a constrained environment for hundreds of vehicles. That is to say, the amount of data generated by cooperative perception can easily lead to the congestion of communication channels, resulting in too much old perception information and reducing the accuracy of perception information [107,108]. Delooz et al. [108] adjusted the network load and optimized the transmission of perceptual objects by filtering the number of objects in the message, including the information about the object itself and low-dynamic objects. However, at the same time, there will be a negative impact on the accuracy of cooperative perception. Thandavarayan et al. [109] demonstrated for the first time that incorporating congestion control functions at the access and facility layers can improve perception through cooperative perception and ensure the timely transmission of information. The combination of the DCC access layer and the facility layer in the simulation scene with a market penetration rate of 100% and high traffic density (180 veh/km), whether using the reactive method or the adaptive method, has significantly improved the object perception rate. Günther et al. [110] evaluated the impact of DCC on cooperative perception in dense scenes and compared the two methods of transmitting environmental CPM using only cooperative awareness messages. They pointed out that a high channel load increases the risk of data packet collision, resulting in low data packet transmission efficiency, increased communication delay, and the reduced accuracy and timeliness of vehicle cooperative perception. Furukawa et al. [111] improved the method of dynamically adjusting the sensor transmission data rate based on the vehicle position relationship and road structure [112]. By autonomously selecting high-probability vehicles to broadcast sensor data, the data of other vehicles’ blind spots covered by sensors can be preferentially transmitted. The method reduces radio traffic and enhances real-time situational awareness of other vehicles. Research on transmission power control has a great effect on the improvement of communication performance. Table 8 describes the possible impact of network performance (delay, packet loss, channel congestion, etc.) on cooperative perception.

**Figure 10:**
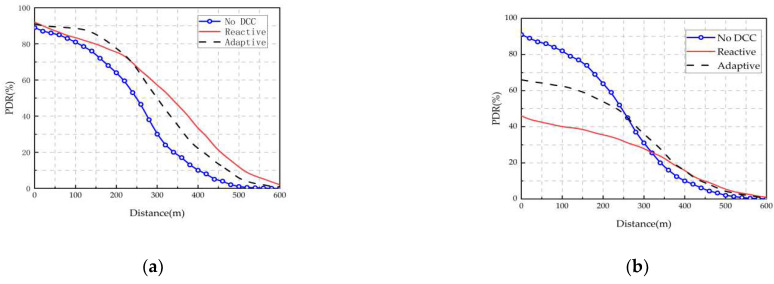
PDR levels for radios and applications with different DCC configurations when all packets have the same priority (DP2). Traffic density: 180 veh/km. (**a**) Radio level. (**b**) Application level.

**Figure 11:**
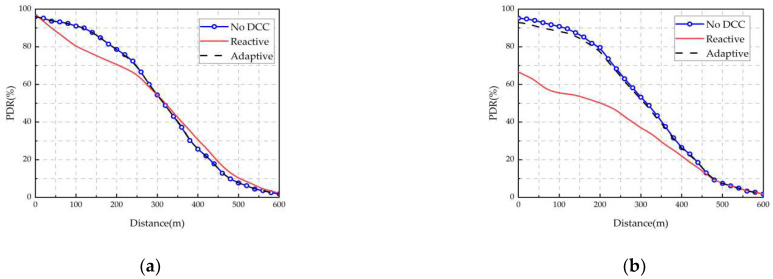
PDR levels for radios and applications with different DCC configurations when all packets have the same priority (DP2). Traffic density:120 veh/km. (**a**) Radio level. (**b**) Application level.

Sepulcre et al. [113] first explored the impact of packet rate-degraded congestion control protocols on the application-level performance of vehicular networks. The results show that the method of controlling vehicle packet loss can reduce the flow of data packets transmitted to the wireless channel and improve communication performance. However, dropped packets are not transmitted, resulting in the reduced performance of the application and a negative impact on the co-awareness effect. As shown in Figure 10 and Figure 11, the radio performance and application-level performance are analyzed for the PLR at traffic densities of 120 veh/km and 180 veh/km, respectively. If the DCC mechanism is not used, both reactive and adaptive methods can improve radio performance, but the application-level performance decreases. The main reason is that application-level packets are not discarded by DCC, as shown in Figure 10. At a traffic density of 120 veh/km, the radio performance is hardly affected, and the application-level performance still drops a large number of packets due to the reactive method, resulting in application-level performance degradation. Furthermore, there is no benefit to using DCC for the application-level evaluation of the packet delivery ratio (PDR) at medium and low traffic densities, as shown in Figure 11.

### 3.4. Summary

To sum up, the share network and information-sharing strategies are key elements in realizing the cooperative perception of autonomous driving in the environment of the IoV. In terms of network communication, DSRC and C-V2X are the key communication methods to realize the cooperative perception of the IoV environment. At this stage, the hybrid network architecture of DSRC and C-V2X can be used to support the diversified needs of IoV applications. Advanced vehicle applications requiring high reliability, low latency, and high throughput will be better supported as communication technology continues to enhance. For the cooperative perception information-sharing strategy, the generation and design of cooperative perception information are further optimized according to the importance and relevance of CPM through the analysis and improvement of standard CPM rules. In addition, factors such as traffic flow, market penetration, communication distance, etc. may cause a series of negative effects, such as long transmission times of cooperative perception information, packet loss, and channel congestion, reducing the effectiveness of cooperative perception. At present, the evaluation indicators of the effectiveness of communication technology and sharing strategies for cooperative perception have not been perfected. In the future, the impact of vehicle mobility and market penetration on communication performance should be fully considered, and a more reasonable CPM-sharing strategy and a more complete evaluation system should be designed.

## 4. Discussion

Autonomous driving cooperative perception technology in the IoV environment greatly solves the current problems of single autonomous vehicles, such as limited perception range and the perception of blind spots. At the same time, the transmission delay is reduced through a new generation of wireless communication technology to ensure the end-to-end timeliness of autonomous driving, from perception to decision-making. However, the particularities of autonomous driving and the maturity of communication technology still pose many problems for cooperative perception. Combined with the existing research and analysis on cooperative perception technology and communication technology, this section further proposes research trends and problems to be solved in V2X and autonomous driving cooperative perception.

### 4.1. Cooperative Perception Information Fusion

The existing image–image, point cloud–point cloud, and image–point cloud perception information fusion methods coexist, and it has been found that multimodal and multi-view image–point cloud fusion cooperative perception is best. The stages of information fusion and sharing are different, which have an important impact on computing speed and perception accuracy. The fusion of processed data can reduce the amount of transmitted data and significantly reduce the network communication load while the fusion of raw sensor data has a better cooperative perception effect, but it will increase the communication burden and has a certain negative impact on the cooperative perception results. Therefore, under the premise of ensuring the performance of target detection, the transmission of low-value data should be reduced as much as possible, and the accuracy and real-time performance of cooperative perception results should be improved. In addition, the dataset construction of vehicle–road collaboration will be a key factor to ensure the research and evaluation of vehicle–road cooperation algorithms.

### 4.2. Efficient and Reliable Information-Sharing Strategy

**Figure 12:**
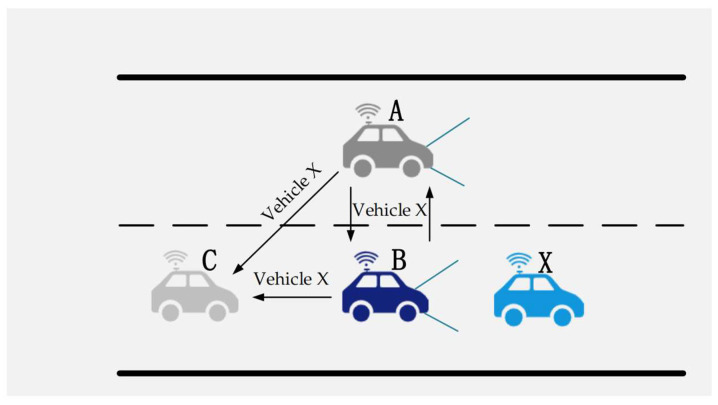
Broadcast-style sharing of cooperative perception data.

Most of the existing cooperative perception information-sharing strategies are implemented in the form of broadcasting [86]. As opposed to unicast and multicast, broadcast messages do not have an acknowledgement mechanism, which not only increases the burden on wireless channels but also makes it difficult to ensure the reliability of perception information transmission when the channel is congested. Perceptual information-sharing is carried out in the form of broadcasting, and the information about the same object may be shared with the autonomous vehicle multiple times, as shown in Figure 12. Vehicle C receives two pieces of information about vehicle X from vehicle A and vehicle B. At this time, vehicle C will perform target matching on the received objects to determine whether they are the same object, which will not only occupy more communication resources but also increase computational tasks for autonomous vehicles. In addition, vehicle C may not need to pay attention to the information of vehicle X so that the path planning and decision-making can be carried out normally. Therefore, proper redundant data sharing helps improve the accuracy of perception, but too much redundant information is obviously not beneficial.

The communication resources of the vehicle network are very precious, so it is necessary to carry out reasonable task scheduling and resource allocation to the edge nodes. Through the reasonable optimization of the CPM-sharing strategy, the information in the area of interest of the vehicle is selectively shared, and the redundancy of data is minimized under the requirements of timeliness and accuracy. At the same time, the deployment of reasonable network technology will effectively reduce the waste of communication resources and realize the sharing of perception information between autonomous vehicles under the constraints of limited communication resources according to different traffic flow, environmental complexity, and vehicle dynamics. Therefore, a reasonable information-sharing strategy will be the focus of future research on cooperative perception.

### 4.3. Vehicle Mobility

The mobility of vehicles is a major problem in the cooperative perception between edge nodes and autonomous vehicles. The highspeed movement of vehicles will lead to the fast movement of communication nodes, frequent changes in network topology, and unstable communication link times, resulting in the frequent connection and disconnection of communication links. Eventually, the link established between V2I and V2V fails, causing the transmission of communication and computing tasks to fail. Secondly, the rapid movement of vehicles will cause rapid changes in the surrounding environment, resulting in varying degrees of interference in the communication between edge nodes and autonomous vehicles. This leads to changes in data transmission efficiency and transmission quality, the degradation of communication quality, and difficulty in ensuring data integrity. In addition, the mission will fail if the vehicle is out of communication range while data uploading or message reception is in progress. At present, the communication quality problem caused by vehicle mobility cannot be effectively avoided. However, the interference of vehicle mobility on communication quality should be minimized to ensure the effectiveness and timeliness of cooperative perception. Zhu et al. [114] considered the frequent changes in the network topology caused by the mobility of vehicles and proposed using regional base stations to uniformly coordinate and manage vehicles within the communication range. When the vehicle with the computing task leaves the communication range, the task scheduling is performed again, which effectively improves the minimum service delay level and service quality. The disadvantage is that additional switching time overhead is required. Zhou et al. [115] utilized a vehicle’s MEC network architecture to seamlessly switch computing tasks through the MEC network routing instead of simply offloading them to edge servers when the vehicle’s communication range changed. This ensures the effective execution of computing tasks and effectively improves the scalability of computing and services. As in [114], it will also increase the time overhead. By introducing a penalty term for the failed tasks in the optimization objective, the task failures caused by vehicle movement can be effectively reduced, but the failures cannot be completely avoided [116,117]. Based on the above research and analysis, it is concluded that the problems caused by vehicle mobility, such as mission failure, poor communication links, and unstable transmission quality, should still be the focus of future research.

### 4.4. Security

The mobility of edge nodes and the dynamics of network topology bring new challenges to the security and confidentiality of vehicle networks. Cooperative perception in the IoV environment mainly solves the information interaction between edge nodes and autonomous vehicles. However, edge nodes, including vehicles, RSUs, and edge servers, are deployed in a distributed manner, resulting in low single-point protection capabilities and attacks by malicious users. When a malicious node sends false environmental information to other vehicles, it sends a large number of computing tasks to the RSU to occupy computing resources and network bandwidth, and the autonomous vehicle that receives the computing tasks will not execute them. The reliability of network services will be destroyed, and the safety of autonomous vehicles will be greatly threatened. Therefore, the realization of autonomous driving cooperative perception in the IoV environment not only needs to consider the trust issue between nodes but also needs to pay attention to the security of the system network. For example, Zhang et al. [118] used elliptic curve cryptography and other security strategies to verify the authenticity of the IoV user access to ensure the security of network communication between vehicles. However, because the IoV is a network without a central node and has no corresponding architecture, coupled with the limited computing resources of the vehicle itself, it is difficult to apply mature traditional network defense solutions to the IoV [119]. The study found that the application of blockchain technology [120,121] can effectively solve the security and privacy protection issues of cooperation between autonomous vehicles and edge nodes under decentralized conditions. Therefore, in the future, it can take advantage of its decentralization, anonymity, and the non-tampering of data to establish a corresponding trust mechanism between edge nodes and autonomous vehicles to solve the trust problem of node data and to reduce network interference.

## 5. Conclusions and Outlook

Cooperative perception technology in the IoV environment plays a crucial role in solving the problems of limited perception range and insufficient computing resources for autonomous vehicles, and through wireless communication technology, it further guarantees the real-time requirements of autonomous driving. This paper introduces the current cooperative perception information fusion methods and cooperative perception information-sharing strategies and sorts out the research methods of cooperative perception information in the process of data transmission under unstable communication conditions. Combined with existing research results, we found that future research on cooperative perception technology should focus on more complex fusion algorithm designs, lightweight models, and V2X applications supported by 5G communication technology. In addition, there are still severe challenges to grasping the balance point of perception–communication–fusion, improving the perception performance of autonomous driving by cooperative perception, and realizing the optimization and balance between V2I and V2V. This paper summarizes the research ideas and analysis methods through a review of the research on autonomous driving cooperative perception in the IoV environment, helping researchers in this field quickly understand the research status of autonomous driving cooperative perception technology in the IoV environment and providing future research directions.

## Figures and Tables

**Table 1:** A comparison of the commonly employed sensors in self-driving cars, only sensors fusion, and combined V2X. The “√” symbol indicates that the sensor operates competently under the specific factor. The “~” indicates that the sensor performs reasonably well under the specific factor. The “×” indicates that the sensor does not operate well under specific factors relative to the other sensors.

Factor	Camera	LiDAR	Radar	Only Fusion	Fusion and V2X
Range	~	~	√	~	√
Resolution	√	~	×	√	√
Distance Accuracy	~	√	√	~	√
Velocity	~	×	√	√	√
Color Perception (e.g., traffic lights)	√	×	×	√	√
Object Detection	~	√	√	~	√
Object Classification	√	~	×	√	√
Lane Detection	√	×	×	√	√
Obstacle Edge Detection	√	√	×	√	√
Illumination Conditions	×	√	√	~	√
Weather Conditions	×	~	√	~	√

**Table 2:** Summary and analysis of image fusion research methods.

Authors, Year	Key Research Points	Findings	Remarks
Lian et al. 2020 [15]	Multiple roadside camera perception data mapping to form a global semantic description.	The detection time was increased by about 45%, and the detection accuracy was increased by about 10%.	Distributed interactive fusion deployment of sensors for a wider range of cooperative perception without increasing the time cost of computing.
Löhdefifink et al. 2020 [21]	Used a lossy learning method for image compression to relieve the pressure on wireless communication channels.	Image compression requirements were high, and late fusion results of segmentation mask cascades were optimal.	The transmission of processed data can effectively reduce the load on the wireless channel.
Lv et al. 2021 [23]	Based on the separation principle of static background and dynamic foreground, the dynamic foreground was extracted, and the static background and dynamic foreground were re-fused by a generative adversarial network.	The processing time of perceptual information was reduced to 27.7% of the original.	
Xiao et al. 2018 [24]	A bird’s-eye view generated by integrating the perception information of other vehicles expanded the perception range, shared the processed image information, and reduced the network burden.	Solved the problem of obstacle occlusion and reduced the transmission of data volume.	Perception range will be affected by communication distance.
Sridhar1 et al. 2018 [25]	Utilized image feature point matching for data fusion to form vehicle cooperative perception with a common field of view.	Fusion of perception information from other vehicles and conversion to its own coordinate system.	Cooperative perception can effectively expand the perception range of vehicles.
Liu et al. 2018 [27]	Used feature point matching to estimate geometric transformation parameters to solve perception blind spots in congestion.	The intersection over union value was increased by 2~3 times.	Effectively solved the obstacle occlusion, but ignored the problem of viewing angle.

**Table 3:** Summary of recent studies on point cloud fusion methods.

Authors, Year	Key Research Points	Findings	Remarks
Chen et al. 2019 [29]	Shared the original point cloud data for the first time, and analyzed the impact of communication cost and the robustness of positioning errors on cooperative perception.	Sparse point cloud negatively affects perception.	Data-level fusion.
Ye et al. 2020 [30]	Fusion of raw sensor data from multiple vehicles to overcome occlusion and sensor resolution degradation with distance.	Fusing sensor data from multiple viewpoints improved perception accuracy and range.	Data-level fusion.
Chen et al. 2019 [31]	A feature-level fusion scheme was proposed, and the tradeoffs between processing time, bandwidth usage, and detection performance were analyzed.	The detection accuracy within 20 m was improved by about 10%.	Feature-level fusion.
Wei et al. 2019 [32]	Integrated the point cloud data of multiple objects, continuously perceiving the position of surrounding vehicles in cases of limited LiDAR perception and V2V communication failure.	Cooperative perception object detection was more stable than LiDAR-only and V2V-only methods.	Feature-level fusion.
Arnold et al. 2020 [34]	Proposed early fusion and late fusion schemes of single-modal point cloud data to more accurately estimate the bounding box of the detection target.	The recall rate of cooperative perception target detection was as high as 95%.	The detection performance of data-level fusion was better than that of decision-level fusion, but the communication quality was poor.

**Table 4:** Summary and classification of research on image–point cloud fusion methods.

Authors, Year	Key Research Points	Findings	Remarks
Jiang et al. 2019 [35]	Used millimeter-wave radar to filter the target and map it to the image to obtain the region of interest, weighted the detection value and estimated value of the two, and improved the perception accuracy.	Effectively detected small targets in foggy weather.	Strong anti-interference ability. However, the detection frequency was low and cannot meet the real-time requirements.
Fu et al. 2020 [43]	A fusion perception method of roadside camera millimeter-wave radar was proposed, and the Kalman filter was used to evaluate the pros and cons of the perception results.	Both horizontal and vertical had better detection results.	No actual deployment.
Wang et al. 2020 [46]	Combined with real road scenes, filtered background objects detected by radar to achieve the automatic calibration of multiple sensors.	Fast and automatic acquisition of roadside perception fusion information.	Attempt to combine depth information to display detection results in 3D boxes.
Saito et al. 2021 [51]	Projected the point cloud data to the pixel coordinate system of the next frame of the point cloud, performed 3D reconstruction, and improved the accuracy of target detection.	Improved target shape recovery rate and discernible distance.	Further adjustments to real-time models and panoramic cameras to expand the fusion range.
Duan et al. 2021 [4]	An image–point cloud cooperative perception system was proposed, which sends the detected objects within the perception range to the vehicle.	Effectively extended the detection range.	A large amount of calculation and poor real-time performance.
Gu et al. 2021 [54]	Utilized point cloud and image concatenation to form a point cloud single-modality mode and a point cloud–image multimodal mode fusion network.	Multimodality for more environmental changes.	Improved the detection accuracy of the road and had good real-time performance.

**Table 5:** Comparison of the advantages and disadvantages of three different fusion methods.

Methodology	Advantages	Disadvantages	Conclusion
Image fusion 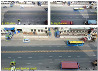	High resolution, richer perception information, good target classification effect, mature algorithm, low deployment cost.	The depth information of the target is insufficient, and it is greatly affected by light and adverse weather.	The image–point cloud fusion scheme has the best effect.
Point cloud fusion 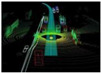	High spatial resolution, rich 3D information, wide detection range, good positioning, and ranging effect.	Poor target classification effect, a large amount of data, easily affected by adverse weather, expensive.	
Image–point cloud fusion 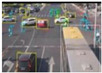	Realizes the complementary advantages of images and point clouds, high resolution, high perception accuracy, richer information, and strong anti-interference.	A large amount of data and a complex algorithm.	

**Table 6:** Summary of the existing research on cooperative perception information-sharing communication technology according to market penetration rate and vehicle mobility.

Factors	Authors, Year	Key Research Points	Findings
Vehicle Mobility	Zhu et al. 2021 [76]	The network architecture of MEC and C-V2X fusion was proposed, which reduces the network transmission delay and improves the reliability of the perception system.	Distributed computing deployment can effectively reduce interaction delay.
	Fukatsu et al. 2019 [77]	Explored the requirements of different driving speeds for network data transmission.	The larger the bandwidth, the better the cooperative perception effect.
	Fukatsu et al. 2021 [78]	Analyzed the data rate required to achieve cooperative perception at different driving speeds.	Derived the transmission data rate for safe driving at different driving speeds.
Traffic Density and Market Penetration	Radovan et al. 2018 [79]	Different sensor and communication equipment deployment schemes will effectively improve the scope of cooperative perception.	Different sensor combinations can make up for the lack of low permeability.
	Li et al. 2021 [80]	Analyzed the impact of market penetration on location and velocity estimates and forecasts.	When the market penetration rate is 50%, the estimated accuracy of vehicle positioning and speed is 80%-90%.
	Wang et al. 2018 [81]	Discussed the capacity requirements for vehicle communication for cooperative perception under different traffic densities and market penetration rates.	V2I traffic from the CPM exchange is highest at about 50% penetration.

**Table 7:** Summary of the perceptual information-sharing strategies by type of shared information.

Sharing Strategy	Authors, Year	Purposes	Findings	Remarks
CPM generation rules	Thandavarayan et al. 2020 [84,85]	Optimized the CPM generation strategy formulated by ETSI to reduce redundant information.	Dynamic CPM generation strategy.	Optimizing the CPM generation strategy can effectively reduce redundant information.
CPM value and freshness	Baldomero et al. 2020 [87]	Designed a context-based confirmation mechanism through which the transmitting vehicle can selectively request the confirmation of specified or critical broadcast information to reduce communication load.	Realized the correct reception of information through message response.	Transmitted critical sensory data, reducing communication load.
	Higuchi et al. 2020 [90]	Decided whether to send the CPM policy by predicting the importance of the CPM to the receiver, reducing the communication load.	Leveraged value prediction networks and assessed the validity of information.	Shared perceptual information based on information importance and freshness.
	Aoki et al. 2020 [91]	Leveraged deep reinforcement learning to select data to transfer.	The detection accuracy was increased by 12%, and the packet reception rate was increased by 27%.	
	Rahal et al. 2020 [92]	Proposed enhancing the freshness of perceptual information to enhance the timeliness and accuracy of cooperative perception information.	Optimized information update rate.	

**Table 8:** Summary of the possible impact of network performance on cooperative perception.

Authors, Year	Key Research Points	Remarks
Liu et al. 2020 [99]	Analyzed the impact of the analysis of factors affecting DSRC performance.	Communication distance and shelter are the main factors that cause the degradation of DSRC communication performance, and selective deployment of roadside equipment can effectively improve DSRC communication performance.
Bae et al. 2021 [100]	Analyzed the impact of communication distance on packet reception rates in LoS and NLoS test scenarios.	Communication distance has a great influence on the reception rate of data packets. The greater the communication distance, the more serious the loss of packet reception rate.
Lee et al. 2020 [102]	Analyzed the impact of PLR and delay on V2X data fusion.	By predicting data changes and using historical data, the accuracy of data fusion can be improved, and the detection accuracy is nearly 50% higher than that of lossy networks.
Xiong et al. 2018 [104]	Evaluated the impact of latency and packet loss on the security of Internet of vehicles applications.	The higher the PLR, the lower the security. The smaller the initial speed, the lower the limit latency.
Thandavarayan et al. 2020 [109]	The study investigated the impact of congestion control on cooperative perception using the DCC framework.	The combination of congestion control functions at the access and facility layers can improve the perception achieved with cooperative perception, ensure the timely transmission of the information, and significantly improve the object perception rate.
Günther et al. 2016 [110]	Selected the best DCC variant and format of messages to maximize vehicle awareness.	The amount of data generated by cooperative perception can easily lead to channel congestion, resulting in too much old sensing information and reducing the accuracy of sensing information.
Furukawa et al. 2019 [111]	Improved the vehicle position relationship and road structure to dynamically adjust the sensor data transmission rate method to improve the transmission rate of useful information.	Selecting high-probability vehicles to broadcast data and prioritizing data from other vehicles’ blind spots reduces radio traffic and enhances the real-time situational awareness of other vehicles.
Sepulcre et al. 2020 [113]	Selected high-probability vehicles to broadcast and prioritize data from other vehicles’ blind spots, reducing radio traffic and enhancing real-time situational awareness of other vehicles.	Controlling the way the vehicle drops packets can reduce the flow of packets transmitted to the wireless channel, but the dropped packets are not transmitted, resulting in the lower performance of the application.

## Data Availability

No new data were created or analyzed in this study. Data sharing is not applicable to this article.

## Abbreviations

The following abbreviations are used in this manuscript:

IoV	Internet of vehicles
V2V	Vehicle-to-vehicle
V2I	Vehicle-to-infrastructure
MEC	Multiaccess edge computing
ROI	Region of interest
CPM	Cooperative perception message
C-V2X	Cellular–vehicle-to-everything
DSRC	Dedicated short range communication
WAVE	Wireless access in vehicular environments
3GPP	Third generation partnership project
ETSI	European Telecommunications Standard Institute
LoS	Line-of-sight
NLoS	Non-line-of-sight
PDR	Packet delivery ratio
DCC	Decentralized congestion control
PLR	Packet loss rate

## References

[1] Marti E., De Miguel M.A., Garcia F., Perez J. (2019). A review of sensor technologies for perception in automated driving. IEEE Intell. Transp. Syst. Mag..

[2] Shuttleworth J. (2019). SAE Standard News: J3016 Automated-Driving Graphic Update. https://www.sae.org/news/2019/01/sae-updates-j3016-automated-driving-graphic.

[3] Yeong D.J., Velasco-Hernandez G., Barry J., Walsh J. (2021). Sensor and sensor fusion technology in autonomous vehicles: A review. Sensors.

[4] Duan X., Jiang H., Tian D., Zou T., Zhou J., Cao Y. (2021). V2I based environment perception for autonomous vehicles at intersections. China Commun..

[5] Lv P., Xu J., Li T., Xu W. (2021). Survey on edge computing technology for autonomous driving. J. Commun..

[6] Wang Z., Wu Y., Niu Q. (2020). Multi-sensor fusion in automated driving: A survey. IEEE Access.

[7] Tesla Driver Killed in Crash with Autopilot Active, NHTSA Investigating. https://www.theverge.com/2016/6/30/12072408/tesla-autopilot-car-crash-death-autonomous-model-s.

[8] Tesla’s Latest Autopilot Death Looks Just Like a Prior Crash. https://www.wired.com/story/teslas-latest-autopilot-death-looks-like-prior-crash/.

[9] Zhang Y., Zhang S., Zhang Y., Ji J., Duan Y., Huang Y., Peng J., Zahng Y. (2020). Multi-modality fusion perception and computing in autonomous driving. J. Comput. Res. Dev..

[10] Ding Z., Xiang J. (2021). Overview of intelligent vehicle infrastructure cooperative simulation technology for IoVs and automatic driving. World Electr. Veh. J..

[11] Mo Y., Zhang P., Chen Z., Ran B. (2021). A method of vehicle-infrastructure cooperative perception based vehicle state information fusion using improved kalman filter. Multimed. Tools Appl..

[12] Lian Y., Qian L., Ding L., Yang F., Guan Y. Semantic fusion infrastructure for unmanned vehicle system based on cooperative 5G MEC. Proceedings of the 2020 IEEE/CIC International Conference on Communications in China (ICCC).

[13] Lv P., He Y., Han J., Xu J. Objects perceptibility prediction model based on machine learning for V2I communication load reduction. Proceedings of the 16th International Conference on Wireless Algorithms, Systems, and Applications (WASA).

[14] Emara M., Filippou M.C., Sabella D. MEC-assisted end-to-end latency evaluations for C-V2X communications. Proceedings of the 2018 European Conference on Networks and Communications (EuCNC).

[15] Ma H., Li S., Zhang E., Lv Z., Hu J., Wei X. (2020). Cooperative autonomous driving oriented MEC-aided 5G-V2X: Prototype system design, field tests and AI-based optimization Tools. IEEE Access.

[16] Rosique F., Navarro P.J., Fernández C., Padilla A. (2019). A systematic review of perception system and simulators for autonomous vehicles research. Sensors.

[17] Li Y., Niu J., Ouyang Z. Fusion strategy of multi-sensor based object detection for self-driving vehicles. Proceedings of the 2020 International Wireless Communications and Mobile Computing (IWCMC).

[18] Jisen W. A study on target recognition algorithm based on 3D point cloud and feature fusion. Proceedings of the 2021 IEEE 4th International Conference on Automation, Electronics and Electrical Engineering (AUTEEE).

[19] Theis L., Shi W., Cunningham A., Huszár F. Lossy image compression with compressive autoencoders. Proceedings of the 5th International Conference on Learning Representations (ICLR).

[20] Xu D., Lu G., Yang R., Timofte R. Learned image and video compression with deep neural networks. Proceedings of the 2020 IEEE International Conference on Visual Communications and Image Processing (VCIP).

[21] Löhdefink J., Bär A., Schmidt N.M., Hüger F., Schlicht P., Fingscheidt T. Focussing Learned Image Compression to Semantic Classes for V2X Applications. Proceedings of the 2020 IEEE Intelligent Vehicles Symposium (IV).

[22] Rippel O., Bourdev L. Real-time adaptive image compression. Proceedings of the International Conference on Machine Learning (ICML).

[23] Lv P., Li K., Xu J., Li T., Chen N. (2021). Cooperative sensing information transmission load optimization for automated vehicles. Chin. J. Comput..

[24] Xiao Z., Mo Z., Jiang K., Yang D. Multimedia fusion at semantic level in vehicle cooperative perception. Proceedings of the 2018 IEEE International Conference on Multimedia & Expo Workshops (ICMEW).

[25] Sridhar S., Eskandarian A. (2019). Cooperative perception in autonomous ground vehicles using a mobile-robot testbed. IET Intell. Transp. Syst..

[26] Kim S.W., Qin B., Chong Z.J., Shen X., Liu W., Ang M.H., Frrazzoli E., Rus D. (2014). Multi-vehicle cooperative driving using cooperative perception: Design and experimental validation. IEEE Trans. Intell. Transp. Syst..

[27] Liu W., Ma Y., Gao M., Duan S., Wei L. (2021). Cooperative visual augmentation algorithm of intelligent vehicle based on inter-vehicle image fusion. Appl. Sci..

[28] Shi J., Wang W., Wang X., Sun H., Lan X., Xin J., Zheng N. Leveraging spatio-temporal evidence and independent vision channel to improve multi-sensor fusion for vehicle environmental perception. Proceedings of the 2018 IEEE Intelligent Vehicles Symposium (IV).

[29] Chen Q., Tang S., Yang Q., Fu S. Cooper: Cooperative perception for connected autonomous vehicles based on 3D point clouds. Proceedings of the 2019 IEEE 39th International Conference on Distributed Computing Systems (ICDCS).

[30] Ye E., Spiegel P., Althoff M. Cooperative raw sensor data fusion for ground truth generation in autonomous driving. Proceedings of the 2020 IEEE 23rd International Conference on Intelligent Transportation Systems (ITSC).

[31] Chen Q., Ma X., Tang S., Guo J., Yang Q., Fu S. F-cooper: Feature based cooperative perception for autonomous vehicle edge computing system using 3D point clouds. Proceedings of the 4th ACM/IEEE Symposium on Edge Computing.

[32] Shangguan W., Du Y., Chai L. (2019). Interactive perception-based multiple object tracking via CVIS and AV. IEEE Access.

[33] Asvadi A., Girão P., Peixoto P., Nunes U. 3D object tracking using RGB and LIDAR data. Proceedings of the IEEE 19th International Conference on Intelligent Transportation Systems (ITSC).

[34] Arnold E., Dianati M., de Temple R., Fallah S. (2020). Cooperative perception for 3D object detection in driving scenarios using infrastructure sensors. IEEE Trans. Intell. Transp. Syst..

[35] Elfring J., Appeldoorn R., Van den Dries S., Kwakkernaat M. (2016). Effective world modeling: Multisensor data fusion methodology for automated driving. Sensors.

[36] Cui Y., Chen R., Chu W., Chen L., Tian D., Li Y., Cao D. (2021). Deep learning for image and point cloud fusion in autonomous driving: A review. IEEE Trans. Intell. Transp. Syst..

[37] Yu Y., Liu X., Xu C., Cheng X. (2021). Multi-sensor data fusion algorithm based on the improved weighting factor. J. Phys..

[38] Jiang Q., Zhang L., Meng D. Target detection algorithm based on MMW radar and camera fusion. Proceedings of the 2019 IEEE Intelligent Transportation Systems Conference (ITSC).

[39] Ji Z., Prokhorov D. Radar-vision fusion for object classification. Proceedings of the 2008 11th International Conference on Information Fusion.

[40] Kocić J., Jovičić N., Drndarević V. Sensors and sensor fusion in autonomous vehicles. Proceedings of the 2018 26th Telecommunications Forum (TELFOR).

[41] Han S., Wang X., Xu L., Sun H., Zheng N. Frontal object perception for intelligent vehicles based on radar and camera fusion. Proceedings of the 2016 35th Chinese Control Conference (CCC).

[42] Zhang X., Zhou M., Qiu P., Huang Y., Li J. (2019). Radar and vision fusion for the real-time obstacle detection and identification. Ind. Robot. Int. J. Robot. Res. Appl..

[43] Zeng S., Zhang W., Litkouhi B.B. (2016). Fusion of obstacle detection using radar and camera. Pat. US.

[44] Jha H., Lodhi V., Chakravarty D. Object detection and identification using vision and radar data fusion system for ground-based navigation. Proceedings of the 2019 6th International Conference on Signal Processing and Integrated Networks (SPIN).

[45] Lekic V., Babic Z. (2019). Automotive radar and camera fusion using generative adversarial networks. Comput. Vis. Image Underst..

[46] Wang X., Xu L., Sun H., Xin J., Zheng N. (2016). On-road vehicle detection and tracking using MMW radar and monovision fusion. IEEE Trans. Intell. Transp. Syst..

[47] Fu Y., Tian D., Duan X., Zhou J., Lang P., Lin C., You X. A camera-radar fusion method based on edge computing. Proceedings of the 2020 IEEE International Conference on Edge Computing (EDGE).

[48] Fan J., Huo T., Li X. A review of one-stage detection algorithms in autonomous driving. Proceedings of the 2020 4th CAA International Conference on Vehicular Control and Intelligence (CVCI).

[49] Şahin M.Ş., Acarman T. An object segmentation approach for mobile lidar point clouds. Proceedings of the 2021 International Conference on Artificial Intelligence and Smart Systems (ICAIS).

[50] Wang L., Zhang Z., Di X., Tian J. A roadside camera-radar sensing fusion system for intelligent transportation. Proceedings of the 2020 17th European Radar Conference (EuRAD).

[51] Saito M., Shen S., Ito T. Interpolation method for sparse point cloud at long distance using sensor fusion with LiDAR and camera. Proceedings of the 2021 IEEE CPMT Symposium Japan (ICSJ).

[52] Lee G.H., Kwon K.H., Kim M.Y. Ambient environment recognition algorithm fusing vision and LiDAR sensors for robust multi-channel V2X system. Proceedings of the 2019 Eleventh International Conference on Ubiquitous and Future Networks (ICUFN).

[53] Lee G.H., Choi J.D., Lee J.H., Kim M.Y. Object detection using vision and LiDAR sensor fusion for multi-channel V2X system. Proceedings of the 2020 International Conference on Artificial Intelligence in Information and Communication (ICAIIC).

[54] Gu S., Yang J., Kong H. A Cascaded LiDAR-camera fusion network for road detection. Proceedings of the 2021 IEEE International Conference on Robotics and Automation (ICRA).

[55] Allig C., Wanielik G. Alignment of perception information for cooperative perception. Proceedings of the 2019 IEEE Intelligent Vehicles Symposium (IV).

[56] Zhang J.W., Liu T.J., Li R.G., Liu D., Zhan J.L., Kan H.W. (2022). A temporal calibration method for multi-sensor fusion of autonomous vehicles. Automot. Eng..

[57] Seeliger F., Dietmayer K. Inter-vehicle information-fusion with shared perception information. Proceedings of the 17th International IEEE Conference on Intelligent Transportation Systems (ITSC).

[58] Rauch A., Klanner F., Rasshofer R., Dietmayer K. Car2x-based perception in a high-level fusion architecture for cooperative perception systems. Proceedings of the 2012 IEEE Intelligent Vehicles Symposium.

[59] Shanzhi C., Yan S., Jinling H. (2020). Cellular vehicle to everything (C-V2X): A review. Sci. Found. China.

[60] Zhou H., Xu W., Chen J., Wang W. (2020). Evolutionary V2X Technologies Toward the Internet of Vehicles: Challenges and Opportunities. Proceedings of the IEEE.

[61] Lu N., Cheng N., Zhang N., Shen X., Mark J.W. (2014). Connected vehicles: Solutions and challenges. IEEE Internet Things J..

[62] Kenney J.B. (2011). Dedicated short-range communications (DSRC) standards in the United States. Proc. IEEE.

[63] Maglogiannis V., Naudts D., Hadiwardoyo S., Akker D.V., Barja J.M., Moerman I. (2022). Experimental V2X evaluation for C-V2X and ITS-G5 technologies in a real-life highway environment. IEEE Trans. Netw. Serv. Manag..

[64] Wei S.G., Yu D., Guo C.L., Shu W.W. (2019). Survey of connected automated vehicle perception mode: From autonomy to interaction. IET Intell. Transp. Syst..

[65] Abdelkader G., Elgazzar K., Khamis A. (2021). Connected vehicles: Technology review, state of the art, challenges and opportunities. Sensors.

[66] Naik G., Choudhury B., Park J.M. (2019). IEEE 802.11bd & 5G NR V2X: Evolution of radio access technologies for V2X communications. IEEE Access.

[67] Abdel Hakeem S.A., Hady A.A., Kim H.W. (2020). 5G-V2X: Standardization, architecture, use cases, network-slicing, and edge-computing. Wirel. Netw..

[68] Bazzi A., Berthet A.O., Campolo C., Masini B.M., Molinaro A., Zanella A. (2021). On the design of Sidelink for cellular V2X: A literature review and outlook for future. IEEE Access.

[69] 5GAA. V2X Technology Benchmark Testing. https://www.fcc.gov/ecfs/filing/109271050222769.

[70] Choi J., Va V., Gonzalez-Prelcic N., Daniels R., Bhat C.R., Heath R.W. (2016). Millimeter-wave vehicular communication to support massive automotive sensing. IEEE Commun. Mag..

[71] Garcia-Roger D., González E.E., Martín-Sacristán D., Monserrat J.F. (2020). V2X Support in 3GPP specifications: From 4G to 5G and beyond. IEEE Access.

[72] Abboud K., Omar H.A., Zhuang W. (2016). Interworking of DSRC and cellular network technologies for V2X communications: A survey. IEEE Trans. Veh. Technol..

[73] Shuguang L., Zhenxing Y. Architecture and key technologies of the V2X-based vehicle networking. Proceedings of the 2019 IEEE 2nd International Conference on Electronics and Communication Engineering (ICECE).

[74] Mir Z.H., Toutouh J., Filali F., Ko Y.B. (2020). Enabling DSRC and C-V2X integrated hybrid vehicular networks: Architecture and protocol. IEEE Access.

[75] Shen X., Li J., Chen L., Chen J., He S. Heterogeneous LTE/DSRC approach to support real-time vehicular communications. Proceedings of the 2018 10th International Conference on Advanced Infocomm Technology (ICAIT).

[76] Zhu X., Yuan S., Zhao P. Research and application on key technologies of 5G and C-V2X Intelligent converged network Based on MEC. Proceedings of the 2021 IEEE International Conference on Power Electronics, Computer Applications (ICPECA).

[77] Fukatsu R., Sakaguchi K. Millimeter-wave V2V communications with cooperative perception for automated driving. Proceedings of the 2019 IEEE 89th Vehicular Technology Conference (VTC2019-Spring).

[78] Fukatsu R., Sakaguchi K. Automated driving with cooperative perception using millimeter-wave V2I communications for safe and efficient passing through intersections. Proceedings of the 2021 IEEE 93rd Vehicular Technology Conference (VTC2021-Spring).

[79] Miucic R., Sheikh A., Medenica Z., Kunde R. V2X Applications using collaborative perception. Proceedings of the 2018 IEEE 88th Vehicular Technology Conference (VTC-Fall).

[80] Li T., Han X., Ma J. (2021). Cooperative perception for estimating and predicting microscopic traffic states to manage connected and automated traffic. IEEE Trans. Intell. Transp. Syst..

[81] Wang Y., De Veciana G., Shimizu T., Lu H. Performance and scaling of collaborative sensing and networking for automated driving applications. Proceedings of the 2018 IEEE International Conference on Communications Workshops (ICC Workshops).

[82] Thandavarayan G., Sepulcre M., Gozalvez J. (2020). Generation of cooperative perception messages for connected and automated vehicles. IEEE Trans. Veh. Technol..

[83] ETSI ITS (2019). Intelligent transport system (ITS); vehicular communications; basic set of applications; analysis of the collective-perception service (CPS). ETSI TR.

[84] Thandavarayan G., Sepulcre M., Gozalvez J. Analysis of message generation rules for collective perception in connected and automated driving. Proceedings of the 2019 IEEE Intelligent Vehicles Symposium (IV).

[85] Thandavarayan G., Sepulcre M., Gozalvez J. Redundancy mitigation in cooperative perception for connected and automated vehicles. Proceedings of the 2020 IEEE 91st Vehicular Technology Conference (VTC2020-Spring).

[86] Rauch A., Klanner F., Dietmayer K. Analysis of V2X communication parameters for the development of a fusion architecture for cooperative perception systems. Proceedings of the 2011 IEEE Intelligent Vehicles Symposium (IV).

[87] Coll-Perales B., Thandavarayan G., Sepulcre M., Gozalvez J. Context-based broadcast acknowledgement for enhanced reliability of cooperative V2X messages. Proceedings of the 2020 Forum on Integrated and Sustainable Transportation Systems (FISTS).

[88] Basagni S., Bölöni L., Gjanci P., Petrioli C., Phillips C.A., Turgut D. Maximizing the value of sensed information in underwater wireless sensor networks via an autonomous underwater vehicle. Proceedings of the 2014 IEEE Conference on Computer Communications (INFOCOM).

[89] Zou P., Ozel O., Subramaniam S. On age and value of information in status update systems. Proceedings of the 2020 IEEE Wireless Communications and Networking Conference (WCNC).

[90] Higuchi T., Giordani M., Zanella A., Zorzi M., Altintas O. Value-anticipating V2V communications for cooperative perception. Proceedings of the IEEE Intelligent Vehicles Symposium (IV).

[91] Aoki S., Higuchi T., Altintas O. Cooperative perception with deep reinforcement learning for connected vehicles. Proceedings of the IEEE Intelligent Vehicles Symposium (IV).

[92] Rahal A.J., Veciana G.d., Shimizu T., Lu H. Optimizing timely coverage in communication constrained collaborative sensing systems. Proceedings of the 2020 18th International Symposium on Modeling and Optimization in Mobile, Ad Hoc, and Wireless Networks (WiOPT).

[93] Talak R., Karaman S., Modiano E. (2019). Optimizing information freshness in wireless networks under general interference constraints. IEEE/ACM Trans. Netw..

[94] Malik R.Q., Ramli K.N., Kareem Z.H., Habelalmatee M.I., Abbas H. A review on vehicle-to-infrastructure communication system: Requirement and applications. Proceedings of the 2020 3rd International Conference on Engineering Technology and its Applications (IICETA).

[95] Noh S., An K., Han W. Toward highly automated driving by vehicle-to-infrastructure communications. Proceedings of the 15th International Conference on Control, Automation and Systems (ICCAS).

[96] Balador A., Cinque E., Pratesi M., Valentini F., Bai C., Gómez A.A., Mohammadi M. (2021). Survey on decentralized congestion control methods for vehicular communication. Veh. Commun..

[97] Xu Q., Pan J.A., Li K.Q., Wang J.Q., Wu X.B. (2021). Design of connected vehicle controller under cloud control scenes with unreliable communication. Automot. Eng..

[98] Chang X.Y., Xu Q., Li K.Q., Bian Y., Han H., Zhang J. (2019). Analysis of intelligent and connected vehicle control under communication delay and packet loss. China J. Highw. Transp..

[99] Liu D.B., Zhang X.R., Wang R.M., Li X.C., Xu Z.G. (2020). DSRC-based vehicle network communication performance in closed field test. Chin. J. Automot. Eng..

[100] Bae J.K., Park M.C., Yang E.J., Seo D.W. (2020). Implementation and performance evaluation for DSRC-based vehicular communication system. IEEE Access.

[101] Xu K., Wang M., Ge Y., Yu R., Wang J., Zhang J. C-V2X Large-scale test network transmission performance data analysis method. Proceedings of the 2021 IEEE 20th International Conference on Trust, Security and Privacy in Computing and Communications (TrustCom).

[102] Lee T.K., Chen J.J., Tseng Y.C., Lin C.K. Effect of packet loss and delay on V2X data fusion. Proceedings of the 2020 21st Asia-Pacific Network Operations and Management Symposium (APNOMS).

[103] Lee T.K., Kuo Y.C., Huang S.H., Wang G.S., Lin C.Y., Tseng Y.C. Augmenting car surrounding information by inter-vehicle data fusion. Proceedings of the 2019 IEEE Wireless Communications and Networking Conference (WCNC).

[104] Xiong G., Yang T., Li M., Zhang Y., Song W., Gong J. A novel V2X-based pedestrian collision avoidance system and the effects analysis of communication delay and packet loss on its application. Proceedings of the 2018 IEEE International Conference on Vehicular Electronics and Safety (ICVES).

[105] ETSI T. (2018). Decentralized congestion control mechanisms for intelligent transport systems operating in the 5 GHz range; access layer part. ETSI TS.

[106] Günther H.J., Riebl R., Wolf L., Facchi C. (2018). The effect of decentralized congestion control on collective perception in dense traffic scenarios. Comput. Commun..

[107] Delooz Q., Festag A. Network load adaptation for collective perception in V2X communications. Proceedings of the 2019 IEEE International Conference on Connected Vehicles and Expo (ICCVE).

[108] Huang H., Fang W., Li H. Performance modelling of V2V based collective perceptions in connected and autonomous vehicles. Proceedings of the 2019 IEEE 44th Conference on Local Computer Networks (LCN).

[109] Thandavarayan G., Sepulcre M., Gozalvez J. (2020). Cooperative perception for connected and automated vehicles: Evaluation and impact of congestion control. IEEE Access.

[110] Günther H.J., Riebl R., Wolf L., Facchi C. Collective perception and decentralized congestion control in vehicular Ad-Hoc networks. Proceedings of the 2016 IEEE Vehicular Networking Conference (VNC).

[111] Furukawa K., Takai M., Ishihara S. Controlling sensor data dissemination method for collective perception in VANET. Proceedings of the 2019 IEEE International Conference on Pervasive Computing and Communications Workshops (PerCom Workshops).

[112] Furukawa K., Takai M., Ishihara S. Controlling sensing information dissemination for collective perception in VANET. Proceedings of the 16th ITS Asia-Pacific Forum.

[113] Sepulcre M., Mira J., Thandavarayan G., Gozalvez J. Is packet dropping a suitable congestion control mechanism for vehicular networks?. Proceedings of the 2020 IEEE 91st Vehicular Technology Conference (VTC2020-Spring).

[114] Zhu C., Tao J., Pastor G., Xiao Y., Ji Y., Zhou Q., Li Y., Antti Y.J. (2018). Folo: Latency and quality optimized task allocation in vehicular fog computing. IEEE Internet Things J..

[115] Zhou S., Netalkar P.P., Chang Y., Xu Y., Chao J. The MEC-based architecture design for low-latency and fast hand-off vehicular networking. Proceedings of the 2018 IEEE 88th Vehicular Technology Conference (VTC-Fall).

[116] Lin C.C., Deng D.J., Yao C.C. (2017). Resource allocation in vehicular cloud computing systems with heterogeneous vehicles and roadside units. IEEE Internet Things J..

[117] Zheng K., Meng H., Chatzimisios P., Lei L., Shen X. (2015). An SMDP-based resource allocation in vehicular cloud computing systems. IEEE Trans. Ind. Electron..

[118] Zhang J.Y., Li F., Li R.X., Li Y.L., Song J.Q., Zhang Q.Y. (2020). Research on identity authentication in V2X communications based on elliptic curve encryption algorithm. Automot. Eng..

[119] Song L., Han Q., Liu J. Investigate key management and authentication models in VANETs. Proceedings of the 2011 International Conference on Electronics, Communications and Control (ICECC).

[120] Han X., Tian D., Sheng Z., Duan X., Zhou J., Long K., Chen M., Leung C.M., Fellow L. (2020). Reliability-aware joint optimization for cooperative vehicular communication and computing. IEEE Trans. Intell. Transp. Syst..

[121] Xu C., Liu H., Li P., Wang P. (2018). A remote attestation security model based on privacy-preserving block-chain for V2X. IEEE Access.

